# TLR2 and TLR4 in Ischemia Reperfusion Injury

**DOI:** 10.1155/2010/704202

**Published:** 2010-06-09

**Authors:** F. Arslan, B. Keogh, P. McGuirk, A. E. Parker

**Affiliations:** ^1^Laboratory of Experimental Cardiology, University Medical Center Utrecht, Heidelberglaan 100, 3584 CX Utrecht, The Netherlands; ^2^Opsona Therapeutics Ltd., Institute of Molecular Medicine, Trinity Centre for Health Sciences, St. James' Hospital, Dublin 8, Ireland

## Abstract

Ischemia reperfusion (I/R) injury refers to the tissue damage which occurs when blood supply returns to tissue after a period of ischemia and is associated with trauma, stroke, myocardial infarction, and solid organ transplantation. Although the cause of this injury is multifactorial, increasing experimental evidence suggests an important role for the innate immune system in initiating the inflammatory cascade leading to detrimental/deleterious changes. The Toll-like Receptors (TLRs) play a central role in innate immunity recognising both pathogen- and damage-associated molecular patterns and have been implicated in a range of inflammatory and autoimmune diseases. In this paper, we summarise the current state of knowledge linking TLR2 and TLR4 to I/R injury, including recent studies which demonstrate that therapeutic inhibition of TLR2 has beneficial effects on I/R injury in a murine model of myocardial infarction.

## 1. Introduction

Our understanding of the molecular components that link dysregulation of innate immunity and human disease has led to a plethora of experimental evidence in support of Toll-like receptors (TLRs) as novel therapeutic targets for a range of inflammatory and autoimmune diseases including rheumatoid arthritis, systemic lupus erythrematosus (SLE), multiple sclerosis, inflammatory bowel disease, cancer and diabetes [1].

TLRs, of which there are currently 10 described in humans, are a family of transmembrane proteins and are the major pattern recognition receptors (PRRs) binding to a range of microbial products, often termed pathogen-associated molecular patterns (PAMPs) [2]. TLR2 functions as a heterodimer with either TLR1 or TLR6 and senses lipopeptides from bacteria, with TLR1/2 dimers recognising triacylated lipopeptides, TLR2/6, and diacylated lipopeptides [2]. TLR4 binds to LPS from gram negative bacteria, which is presented to TLR4 by the accessory factor MD2 [2]. TLR4 can also recognise F protein from respiratory syncytial virus and glycerophosphatidylinositol anchors from parasites and TLR5 binds bacterial flagellin. TLR3 senses double-stranded RNA, TLR7/8 both recognise single-stranded RNA, and TLR9 senses CpG-rich hypomethylated DNA [2]. 

On activation of the receptor, adaptor molecules (MyD88, MAL, TRIF and TRAM) are recruited to the receptor through their respective TIR domains, which interact with the TIR domain of the TLR. This allows the recruitment and activation of a downstream family of kinases, IRAKs (IL-1 receptor-associated kinases) 1, 2, and 4. IRAK4 is recruited to the complex first, becomes activated, and phosphorylates IRAK1. These kinases lead to activation of further downstream kinases, including inhibitor of NF-*κ*B (I*κ*B) kinases (IKKs), resulting in the release of NF-*κ*B from I*κ*B, allowing NF-*κ*B to translocate to the nucleus and mediate an increase in inflammatory cytokine gene expression, leading to pro-inflammatory responses [2, 3].

 In addition to the recognition of PAMPs, TLR2, TLR4, and TLR9 have also been shown to recognise endogenous ligands, which have been termed danger-associated molecular patterns (DAMPs). TLR2 and TLR4 are extracellular TLRs and have a wide range of putative endogenous ligands which include heat shock proteins, high mobility group box 1 (HMGB1) and breakdown products of fibronectin, heparan sulfate, and hyaluronic acid. The broad expression profile of the TLRs and their ability to recognise many ligands that are released predominantly as a consequence of injury and stress positions TLR dependent signaling as a rapid response mechanism to local tissue damage.

## 2. Ischemia Reperfusion Injury

There is a growing body of evidence linking TLRs, particularly TLR2 and TLR4, to the deleterious inflammatory effects seen in ischemia/reperfusion injury associated with trauma, stroke, myocardial infarction, and solid organ transplantation. Ischemia reperfusion injury refers to the tissue damage caused when blood supply returns to tissue after a period of ischemia. Cessation of arterial blood flow with immediate oxygen deprivation of cells (ie, hypoxia with accumulation of metabolic products) is defined as ischemic injury. Tissue can be subjected to periods of either cold or warm ischemia depending on the clinical setting. Cold ischemia occurs typically in transplantation after organ harvesting and static cold preservation whilst warm ischemia occurs during vascular anastomosis or following organ traumas such as stroke or myocardial infarction. The resistance of various cell populations to different types of ischemia varies depending on the affected organ, for example, cardiac endothelial cells are quite resistant to warm ischemia, and major endothelial injury is only apparent during the reperfusion phase [4]. In contrast, hepatocytes and Kupffer cells in the liver and kidney proximal tubular cells are extremely sensitive to periods of warm ischemia [5, 6]. Nevertheless organs can tolerate prolonged cold ischemia periods or short periods of warm ischemia without significant deterioration of function. However, when ischemia is followed by reperfusion, significant cellular damage is caused [7] which in the case of solid organ transplantations has been associated with an increased incidence of delayed graft function (DGF) and primary graft nonfunction [8, 9].

The absence of oxygen and nutrients from blood creates a condition in which the restoration of circulation (reperfusion) through the ischemic tissue results in a set of reactions that can cause injury to vascular and parenchymal cells [10]. Although the cause of this injury is multi-factorial, increasing experimental evidence suggests an important role for the innate immune system in initiating the inflammatory cascade leading to detrimental/deleterious changes. Pathologically, reperfusion-induced inflammation is characterised by deposition of complement, upregulation of adhesion molecules, inflammatory cell infiltration, and cytokine release [11–14]. Neutrophils, monocytes and lymphocytes are the principal immune cells implicated in this process [15, 16] and facilitate tissue damage through secretion of proinflammatory cytokines, reactive oxygen species, and chemokines.

## 3. Cardiac Ischemia/Reperfusion

Cardiac ischemia/reperfusion is predominantly associated with myocardial infarction but can also be seen in transplant and coronary artery bypass graft (CABG) surgery. Early reperfusion of the ischemic myocardium is a prerequisite for cardiomyocyte salvage in myocardial infarction. The shorter the ischemic period, the better the clinical outcome. Postischemic reperfusion causes deleterious responses in both cardiomyocytes and circulating cells [17]. Myocardial I/R (MI/R) injury is the acceleration of both apoptosis and necrosis of cardiomyocytes at the onset of reperfusion therapy, resulting in an increase of infarct size, arrhythmias and contractile dysfunction [18–22]. Current reperfusion therapy remains suboptimal and necessitates adjunctive interventions to limit infarct size and enhance clinical outcome. Experimental studies have clearly demonstrated that infarct size can be reduced when MI/R injury is prevented [20, 22]. 

Reperfusion after myocardial ischemia is a typical “double-edged sword” that results in disease specific changes within cardiomyocytes and circulating cells [23, 24]. The fact that characteristic pathological changes occur in these two compartments (parenchymal, i.e., cardiomyocytes versus. hematopoiesis-derived cells) creates the opportunity to tackle MI/R injury in two ways; either enhance cardiomyocyte survival and/or disarm deleterious circulating cells. Interestingly, TLRs are expressed in both compartments; leukocytes, endothelial cells [25] and cardiomyocytes [26] express certain TLRs that can have different pathophysiological consequences for both the host cell as well as distant organs. Upon ligand binding (either PAMPs or DAMPs), cardiomyocytes and endothelial cells undergo the same TLR signal transduction compared to leukocytes. The main hurdle in MI/R injury is a positive feedback loop between inflammation and cardiomyocyte death: leukocyte-cardiomyocyte/endothelial cell interaction causes cardiomyocyte death, which in turn releases the same cytokines that activate and attract leukocytes [24]. Recent studies using murine MI/R injury models reveal that both TLR2^(−/−)^ and TLR4^(−/−)^ offer protection from this vicious circle resulting in a decrease of infarct size and improved cardiac function [27].

Within the first few minutes after reperfusion, NF-*κ*B translocates to the nucleus to induce pro-inflammatory and proapoptotic gene expression promoting cell dysfunction and death [24]. Interestingly, experimental data indicate that parenchymal (i.e. myocardium, endothelium) and circulatory TLR2 are associated with different manifestations of MI/R injury. MI/R injury has four manifestations that are associated with worse cardiac function and clinical outcome. Lethal MI/R injury is directly related to cell death and responsible for infarct size increase during reperfusion. The so-called “no-reflow phenomenon” is the disturbance of coronary flow in the culprit coronary artery after reperfusion [28]. Stunning is contractile dysfunction of the myocardium in the presence of restored coronary flow [29] and reperfusion associated arrhythmias [30]. Investigators have observed the different manifestations of MI/R injury, either deliberately or by accident, by using several challenging experimental methods. TLRs are expressed by both compartments and therefore the relative contribution of parenchymal and circulating TLRs to a certain disease entity requires the use of chimeric mice (WT mice transplanted with knockout bone marrow and vice versa). 

Sakata et al. were among the first to show a critical role for TLR2 in an ex vivo model for MI/R injury [31]. Without the detrimental effects of blood components (e.g. leukocytes) after reperfusion, they showed that infarct size did not differ between TLR2^(−/−)^ and WT hearts. Nevertheless, contractile performance was significantly impaired in WT hearts, and associated with increased levels of TNF*α* and IL-1*β* in the myocardium. These data indicate that in the presence of cardiac ischemic injury, loss of cardiac TLR2 signaling is beneficial for cardiac function. The direct effect of TLR2 activation on contractile performance has been confirmed in an in vitro setting using a murine cardiomyocyte cell line. Boyd et al. showed that stimulation of TLR2 resulted in decreased contractility of plated cardiomyocytes [26]. The finding that TLR2^(−/−)^ mice have preserved contractile performance compared to WT mice in the setting of *S. aureus* induced sepsis, serves as a proof for the link between cardiac TLR2 activation and depressed cardiac function.

Endothelial dysfunction as seen in “no-reflow” seems to be mediated by both endothelial and circulating TLR2. Impaired relaxation responses after MI/R injury were observed in isolated coronary arteries of WT mice and TLR2^(−/−)^ mice with WT bone marrow [32]. The fact that both compartments play a role in endothelial dysfunction after MI/R injury indicates that, indeed, a vicious circle caused by the interaction of leukocytes and endothelial cells is critical in MI/R injury-related endothelial dysfunction.

We and others were the first to document decreased infarct size in TLR2^(−/−)^ mice [32, 33]. Using chimeric TLR2^(−/−)^ mice, we showed that circulating TLR2 completely mediated TLR2-dependent lethal MI/R injury. Infarct size in WT mice with TLR2^(−/−)^ bone marrow was similar to that in complete knockouts. In addition, TLR2^(−/−)^ mice with WT bone marrow were not protected at all against MI/R injury, suggesting that parenchymal (i.e. cardiac/endothelial) TLR2 signaling does not play a role in lethal MI/R injury. Systemic administration of a TLR2 antagonist just prior to reperfusion, thus inhibiting circulating TLR2 activation, decreased infarct size and improved cardiac function via downregulated inflammation and apoptotic signaling in mice [33]. 

Ischemic preconditioning (IPC) is a phenomenon in which brief episodes of repeated ischemia protects the heart against a more severe and prolonged period of ischemia and subsequent reperfusion [34]. In line with the above mentioned findings, TLR2 also appears to play a pivotal role in IPC. TLR2^(−/−)^ hearts subjected to MI/R injury did not benefit from IPC, whereas TLR4^(−/−)^ hearts did show improved contractile function after IPC and subsequent MI/R injury [35].

## 4. Renal Ischemia/Reperfusion

Renal ischemia/reperfusion is most commonly associated with either trauma or transplant. It is possible to detect mRNA for all TLRs in human kidney but TLR2 and TLR4 have been the TLRs primarily implicated in mediating renal ischemia/reperfusion injury. TLR2 and 4 are constitutively expressed in both proximal and distal tubules, the thin limb of the loop of Henle and the collecting ducts. Expression is upregulated in these areas post I/R [36]. Several studies using TLR2^(−/−)^ and TLR4^(−/−)^ mice have demonstrated a protective effect in models of renal I/R. Leemans et al. [37] used both KO mice and antisense oligonucleotides to show that blockade of TLR2 has a beneficial effect on renal I/R injury. Following I/R, TLR2^(−/−)^ mice displayed less tubular epithelial apoptosis, a reduced cellular infiltrate and reduced dysfunction. Through the generation of chimeric mice, the authors also showed that TLR2 expressed on renal parenchyma was the key cell type involved in renal tissue injury which is in contrast to the findings in myocardial infarction model where neutrophils and monocytes were the key cell types mediating injury [33]. Wu et al. [38] demonstrated up-regulation of TLR4 in tubular epithelial cells in response to renal I/R and protection against kidney dysfunction in TLR2^(−/−)^ and Myd88^(−/−)^ mice. They also showed up-regulation of HMGB-1, hyaluronan and brevican, all proposed ligands for TLR2 and TLR4. Pulskens et al. [39] carried out a similar study using TLR4^(−/−)^ mice demonstrating a protective effect on renal function, chemokine production and cellular infiltration. 

The molecular pathways involved in TLR2-mediated damage in renal I/R were investigated by Shigeoka et al. [40]. Using a range of transgenic mice, this study showed that TLR2^(−/−)^ mice were better protected from I/R damage than those deficient in MyD88, indicating that pathways dependent on TLR2 but independent of MyD88 contribute to kidney injury. More recently Rusai et al. [41] compared TLR2^(−/−)^ and TLR4^(−/−)^ mice with the double knockout TLR2/4^(−/−)^ demonstrating protective effects with both single knock-outs but surprisingly no increased protection when both TLR2 and TLR4 are deleted. This may indicate that TLR2 and TLR4 prime each other in the presence of endogenous ligands, such as during reperfusion injury. 

There is also a body of evidence suggesting that TLR2 plays a role in transplantation tolerance. Wang et al. have demonstrated the important role of TLR and TLR signalling pathways in the pathogenesis of kidney chronic allograft dysfunction [42]. In this paper, TLR2 and MyD88, deficiencies significantly improved the excretory function of chronic kidney grafts by 65% and 290% respectively, and histopathologic signs of chronic allograft damage were significantly ameliorated. T cells, dendritic cells (DCs), and macrophages were reduced in grafts by up to 4.5-fold and intragraft concentrations of IL-6, IL-10, monocyte chemotactic protein-1 (MCP-1) and IL-12p70 were significantly lower. In addition TLR2^(−/−)^, MyD88^(−/−)^,and TRIF^(−/−)^ deficient recipients showed a significant reduction in fibrosis with collagen I and III levels reduced by up to twofold compared to wild type mice [42]. These findings highlight the functional relevance of TLRs and their two major signaling pathways in graft infiltrating in the pathophysiology of kidney chronic allograft dysfunction.

## 5. Transplantation

In a cardiac transplantation study, Chen et al. have demonstrated that TLR2 ligation by the TLR1/2 ligand Pam_3_CSK_4_ prevents heart allograft acceptance in mice cotreated with anti-CD154 costimulation therapy. In contrast, mice receiving anti-CD154 treatment alone were observed to display allograft acceptance [43]. In a further study by Jiang et al., innate immunity-mediated cardiac allograft rejection was not prevented by cyclosporine A (CsA) treatment [44]. In this study, mice co-treated with CsA and an anti-inflammatory compound called Serp-1 exhibited a significant down-regulation of TLR2 and TLR4, reduced graft infiltration of macrophage and T lymphocytes posttransplantation and associated indefinite graft survival. In contrast, CsA monotherapy did not prevent TLR2 and TLR4 down-regulation and was ultimately unsuccessful in preventing graft rejection [44]. Interestingly, the chronic application of agents such as cyclosporine, which are currently under investigation in transplant biology, has been associated with renal injury, which has been correlated with upregulation of TLR2 expression on renal tubular cells [45]. Furthermore, increased angiotensin II levels following CsA administration have also been shown to directly upregulate TLR2 expression [46]. 

Delayed Graft Function (DGF) is a frequent consequence of reperfusion injury of the transplanted donor organ. Furthermore, organ shortages and increased patient waiting times for transplants has necessitated the use of extended criteria and nonheart beating donors. Organs from these donors are exposed to periods of both warm and cold ischemia and as such are significantly more susceptible to DGF than organs from heart beating donors which are only exposed to periods of cold ischemia. Krüger et al. [47] looked at expression levels of TLR4 in human kidney transplants. Whilst TLR4 is constitutively expressed in donor organs, the level of expression was significantly higher in non-heart beating donor kidneys which also correlated with increased levels of HMGB-1. They also genotyped the organs for known TLR4 loss of function mutations which alter signalling in response to HMGB-1 and other ligands. Those organs carrying TLR4 mutations exhibited reduced levels of cytokines and a higher rate of immediate graft function. This argues that there is a significant donor TLR4 effect contributing to inflammation and graft function following cold ischemia and transplantation. The role of HMGB-1 has also been explored in a murine model of heart transplant where administration of a neutralising antibody to HMGB-1 reduced levels of circulating cytokines [48]. 

Jiang et al. have recently shown that in a rodent model of liver transplantation the expression of mRNA and protein of TLR2 and TLR4, CD14 and MD-2 mRNA as well as endogenous ligands of TLR2 and TLR4 such as HSP60 and HSP70 were quickly and significantly increased after reperfusion, and reached a peak at 3 h after reperfusion. The appearance of TLR2 and TLR4 mRNA was accompanied by increased HSP 60 and 70 mRNA within 24 h after reperfusion [49]. In addition to these studies, CD14^+^TLR2^+^ monocytes have been demonstrated to be significantly upregulated in patients with acute liver transplant rejection but not in those with normal liver function post transplantation [50]. Stimulation of TLR2 has also been associated with acute skin graft rejection in a murine co-stimulation blockade model where successful skin grafts were observed with anti-CD154 treatment alone but not in the presence of a TLR2 agonist [51]. In contrast, TLR2-defective animals' exhibit prolonged skin graft acceptance [52]. In the latter model, it is worth noting that it is now well-established that lung, intestine and skin are more susceptible to acute rejection episodes posttransplantation than kidney, heart, and pancreas [53]. These studies serve to illustrate further the potential of TLR2 blockade in solid organ transplantation and predict a successful outcome in the amelioration of organ dysfunction post transplant.

## 6. Other I/R Settings

Several studies have indicated a role for TLR2 in ischemia of other organs such as brain [54–56], liver [57–59], bowel [60], and kidney [36, 61–63]. These studies highlight the complexity of the role of TLR signaling in ischemia/reperfusion.

Zhang et al. [59], showed that TLR2 mRNA was increased in the ischemic lobes of mice that underwent partial hepatic I/R. This was associated with an increase in TNF-*α* in the portal vein, and was independent of endotoxemia as portal vein endotoxin did not increase. This indicates a potential role for endogenous TLR2 ligands in liver I/R. Shen et al. [58] used TLR2^(−/−)^ and TLR4^(−/−)^ mice to elucidate the role of TLRs in liver ischemia. They showed that in warm I/R in the liver, hepatocellular injury in WT and TLR2^(−/−)^ mice was equally severe. This was associated with increased TNF-*α* and TLR4. However, when TLR4 signaling was prevented, hepatic injury was ameliorated and this was associated with reduced TNF-*α* levels. There was no effect on expression of TLR2. These effects appeared to be dependent upon intrahepatic expression of heme oxygenase 1 (HSP32). The role of HSPs, in this case HSP72, was further investigated by Galloway et al. [57]. This study looked at ex vivo hepatocytes from TLR2 and TLR4^(−/−)^ mice stimulated with HSP72. HSP72 induced MIP-2 in WT cells, this was ablated in TLR2^(−/−)^ and TLR4^(−/−)^ mice. Interestingly, no effect on the concentration of either IL-6 or TNF was seen. The importance of TLR2 and TNF-*α* was also shown by Zhang et al. [64]. However, while this study showed that a decrease in TNF-*α* concentration was beneficial, they also showed that a decrease in TLR2 expression correlated with a benefit on ischemic outcome. Hui et al. used a model of hepatic I/R and chimeric bone marrow TLR4^(−/−)^ mice to demonstrate that protective effects were provided by both parenchymal and circulating cells [65]. Jin et al. [66] used N-acetylcysteine (NAC) to prevent the generation of reactive oxygen species (ROS) in a mouse model of hepatic I/R. They showed that TLR2 and 4 were activated in the liver and lung. It appears that ROS increases NF-*κ*B activity and causes its translocation to the nucleus. NAC inhibited the activation of TLR2 and 4, and the associated induction of TNF-*α*. 

TLR2 and TLR4 both appear to play critical but opposing roles in cerebral ischemia. Tang et al. [55] recently showed that both TLR 2 and 4 are expressed in cerebral cortical neurons and that selective elimination of their function suppresses activation of JNK. This protects neurons against death by energy deprivation and stroke. This indicated that TLRs also play a role in cerebral ischemia. Ziegler et al. [54] compared the response of TLR2^(−/−)^ and TLR4^(−/−)^ mice to cerebral ischemia. They confirmed earlier findings that TLR2 is indeed up-regulated in cerebral ischemia. However, contrary to Hua et al. [67], they showed that TLR2^(−/−)^ mice had a smaller infarct size. In an earlier study, Hua et al. [68] showed that preconditioning with Pam3Csk4, a TLR2 agonist, 24 hours prior to 1 hour of cerebral ischemia significantly reduced brain infarct size, possibly via an effect on blood brain barrier integrity. In a later study [67], the same authors used knockout mice to show differential roles for TLR2 and TLR4 in cerebral ischemia. This study showed that brain infarct size was significantly less in TLR4^(−/−)^ mice but was increased in TLR2^(−/−)^ mice. This was associated with activation of the PI3K/AKT pathway in TLR4^(−/−)^ mice. This pathway was inhibited in TLR2^(−/−)^ mice. The difference between this study and that of Ziegler et al. [54] may be explained by the fact that Zeigler et al., occluded the middle cerebral artery, whereas Hua et al. [67] occluded the common carotid artery and the internal carotid artery or alternatively may be a consequence of the differing genetic backgrounds of the transgenic mice.

## 7. Conclusions and Perspective

TLRs play complex roles in I/R injuries and the wealth of data generated using transgenic mice is not always in agreement. Both TLR2 and TLR4 appear to be key regulators on the outcome of ischemic damage in many organs. The relative contribution of parenchymal cells and leukocytes to I/R injury and subsequent inflammation appears to differ between organs and manifestations of reperfusion injury. Where double knockouts have been generated, it is perhaps surprising that there is no synergistic effect where each single knockout provides improvement in function and inflammation. There are also conflicting reports of the relative importance of Myd88-dependent and -independent signaling in conferring protection from I/R injury. 

The specific benefit of inhibiting TLR responses appears to be governed by the organ however it remains to be established what the triggers and/or activators are for I/R injury. From a “danger model” perspective [69], it is postulated that molecules released during cell stress or cell death may serve as endogenous ligands for TLRs in ischemia [33]. In essence, the entire disrupted milieu within apoptotic cells represent potential candidate ligands when one considers that all hydrophobic portions (so called “Hyppos”) of molecules may initiate a danger signal [70]. So far, a few specific danger-associated molecules (e.g. HMGB1, HSP60, cardiac myosin) have been postulated as TLR ligands in ischemia largely based upon increased levels in damaged tissue but direct in vivo evidence that these drive the inflammatory response is still lacking. Studies on knockout animals have been very informative and clearly established a central role for TLR2 and TLR4 in mediating I/R injury. Nevertheless, therapeutic intervention studies in both small and large animal models are required to fully understand the potential of TLR signaling as pharmaceutical targets. The tools are now available to inhibit TLR2- and TLR4- dependent signaling in mice as well as neutralizing antibodies to postulated ligands such as HMGB-1. In addition, further work is required in other organs such as liver and brain to better understand if agonism or antagonism is required to confer the benefit. 

Recent studies clearly demonstrate that inhibition of TLR2 has beneficial effects on I/R injury in a murine model of myocardial infarction [33] offering the first evidence that therapeutic benefit can be derived from targeting TLR2. Further studies are required to expand upon these observations and determine the potential for both TLR2 and TLR4 antagonists in treating I/R injury in multiple organs.

## Figures and Tables

**Figure 1:**
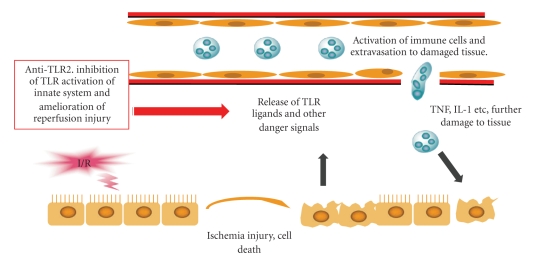
Ischemia–reperfusion injury is characterized by a sublethal injury to epithelial cells resulting in the release of TLR activating danger signals. These danger signals promote the production of chemokines, cytokines, oxygen free radicals and the extravasation of leucocytes from the circulation that amplifies cell damage. Compounds which block TLR activation represent a novel therapeutic mechanism to inhibit this pro-inflammatory cascade thus reducing I/R damage and improving organ function.
